# Determinants of Birth Asphyxia Among Newborns at a Tertiary Care Hospital, Central Ethiopia: A Case‐Control Study

**DOI:** 10.1155/bmri/5402567

**Published:** 2025-11-19

**Authors:** Terefe Alemayehu, Nesra Mohammed Fati, Abebe Megerso Adlo, Alem Deksisa, Anteneh Tefera Chirnet, Elias Bekele Wakwoya, Yohannes Mekuria Negussie

**Affiliations:** ^1^ Department of Anesthesia, Adama Hospital Medical College, Adama, Ethiopia; ^2^ Department of Biomedical Sciences, Adama Hospital Medical College, Adama, Ethiopia; ^3^ Department of Public Health, Adama Hospital Medical College, Adama, Ethiopia; ^4^ Department of Nursing and Midwifery, Arsi University, Asella, Ethiopia, arsiun.edu.et; ^5^ Department of Medicine, Adama General Hospital and Medical College, Adama, Ethiopia

**Keywords:** birth asphyxia, case-control, determinants, Ethiopia, newborn

## Abstract

**Background:**

Birth asphyxia is a major cause of newborn deaths worldwide, especially in developing countries where access to skilled delivery care is limited. It is a significant health challenge in Ethiopia, contributing to many newborn deaths and long‐term health issues. Despite efforts to improve maternal and newborn care, it remains a serious concern. Thus, this study was aimed at identifying the determinants of birth asphyxia among newborns at a tertiary care hospital in Central Ethiopia.

**Methods:**

An institution‐based unmatched case‐control study was conducted among 345 participants with a case‐to‐control ratio of 1:2. Data were collected using a pretested, structured, interviewer‐administered questionnaire and a data abstraction checklist. The collected data were entered into Epi Info Version 7.2 and analyzed using SPSS Version 27. Binary logistic regression analysis was performed to identify the determinants of birth asphyxia. Adjusted odds ratios (AORs) with 95% confidence intervals (CIs) were used to estimate the strength of associations. Statistical significance was set at a *p* value < 0.05.

**Result:**

In this study, the place of residence (rural) (AOR = 2.34; 95% CI: 1.29–4.26), premature rupture of membrane (AOR = 3.47; 95% CI: 1.52–7.92), prolonged labor (AOR = 10.12; 95% CI: 5.36–19.11), noncephalic fetal presentation (AOR = 2.40; 95% CI: 1.01–5.74), instrumental delivery (AOR = 2.67; 95% CI: 1.15–6.16), and cesarean section delivery (AOR = 3.99; 95% CI: 1.84–8.63) were identified as independent determinants of birth asphyxia.

**Conclusion:**

Rural residence, premature rupture of membranes, prolonged labor, noncephalic fetal presentation, instrumental delivery, and cesarean section delivery were determinants of birth asphyxia. Efforts to reduce birth asphyxia should focus on improving maternal healthcare in rural areas, enhancing the management of labor complications, and ensuring skilled delivery care, especially for noncephalic presentations and operative deliveries.

## 1. Introduction

Birth asphyxia occurs when a newborn cannot begin or keep breathing properly [1, 2]. It is marked by difficulties in exchanging oxygen and carbon dioxide, leading to low oxygen levels (hypoxemia), high carbon dioxide levels (hypercapnia), and an imbalance in the body′s acid levels (metabolic acidosis) [2]. The World Health Organization (WHO) defines it as “the failure to initiate and sustain breathing at birth” [3]. This condition arises when the brain and other organs do not receive enough oxygen and nutrients, and it can happen before, during, or after birth [2, 4].

Birth asphyxia is more common in developing countries compared to developed ones, primarily due to limited access to skilled care during delivery [5]. Globally, it affects two to 10 out of every 1000 full‐term newborns, with higher rates in developing nations [6]. In low‐ and middle‐income countries, birth asphyxia is a leading cause of newborn deaths and contributes to long‐term health problems such as intellectual disabilities, cerebral palsy, and other developmental disorders [2, 7, 8]. In Africa, the prevalence of birth asphyxia is 15.9%, with East Africa reporting 18% and Central Africa 9.1%. [9]. In Ethiopia, the pooled prevalence is higher, at 19.3% [5].

It accounts for approximately 900,000 deaths annually and is responsible for 23% of all newborn deaths worldwide [3, 10]. In Ethiopia, it is the second leading cause of newborn deaths, with a mortality rate of 26.7% among newborns and 11.3% among children under five [11]. To address this issue, the United Nations set the Sustainable Development Goals in 2015, aiming to reduce newborn deaths to fewer than 12 per 1000 live births by 2030. In line with this, the Ethiopian government has introduced various measures, such as enhancing emergency care for mothers and newborns. However, despite these efforts, newborn mortality remains a significant public health concern [12, 13].

Although numerous factors contribute to birth asphyxia, they are primarily classified into maternal, fetal, and fetomaternal categories [14]. Previous studies have identified several factors contributing to birth asphyxia, including maternal age, educational status, marital status, antenatal care (ANC) follow‐up, parity, gravidity, fetal presentation, premature rupture of membranes, birth weight, gestational age, duration of labor, and mode of delivery [5, 6, 15–20].

To prevent birth asphyxia, reduce neonatal mortality, and improve quality of life, it is essential to identify and address its risk factors as early as possible. Additional studies in diverse settings are necessary to better understand the factors associated with birth asphyxia and mitigate its impact. However, data on the determinants of birth asphyxia in the study area remain limited. Thus, this study was aimed at identifying the determinants of birth asphyxia among newborns at a tertiary care hospital in Central Ethiopia. Gaining insight into the determinants of birth asphyxia is key to formulating comprehensive strategies for its prevention and management. The findings will also contribute to improving healthcare providers′ and women′s knowledge of birth asphyxia during labor and provide context‐specific data to guide interventions aimed at preventing birth asphyxia.

## 2. Methods

### 2.1. Study Design, Area, and Period

A hospital‐based unmatched case‐control study was conducted at Adama Hospital Medical College (AHMC) from January 1 to February 28, 2023. The hospital is located in Adama City, Oromia Regional State, 99 km southeast of Addis Ababa. It serves the community by providing both patient care services and educational programs. As the largest health facility in the East Shewa Zone, the hospital serves more than 6 million people from surrounding regions. The hospital′s gynecology and obstetrics unit handles more than 5000 deliveries annually on average.

### 2.2. Study Population and Eligibility Criteria

All newborn live births delivered at AHMC were considered the source population. The study population consisted of all newborn live births delivered at the hospital during the study period. Newborns with major congenital anomalies, as well as newborns whose mothers (or caregivers) were either lost or deceased, were excluded from the study. Additionally, mothers who were too ill to respond were also excluded.

Operational definitions:

Prolonged labor: active labor lasting more than 12 h, consistent with WHO guidelines.

Premature rupture of membranes (PROMs): rupture of membranes before the onset of labor.

Cases: newborns delivered after viability who exhibited at least one of the following signs: gasping, not breathing, a breathing rate below 30 bpm, or a 5th‐min APGAR score of less than 7.

Controls: newborns who showed no signs of breathing difficulty and had a 5th‐min APGAR score of 7 or greater.

### 2.3. Sample Size and Sampling Procedure

The sample size was determined using Epi Info Version 7.2 statistical software for the unmatched case‐control study. Calculations were based on 80% power, a two‐sided 95% confidence level, a 1:2 case‐to‐control ratio, and an additional 10% contingency to account for nonresponse. Various factors significantly associated with the outcome variable were considered, and the largest required sample size was chosen. Among these determinants, the exposure variable of prolonged labor gave the largest sample size, resulting in a sample of 329 (110 cases and 219 controls). After adding a 10% contingency for nonresponse, the final sample size for this study was 366 (122 cases and 244 controls) (Table 1).

**Table 1:** Sample size determination using the Epi Info 7.0 StatCalc program.

**Variables**	**Percentage of controls exposed to the risk factor**	**Adjusted odds ratio**	**Sample size**	**Total sample size**	**Final sample size after 5% contingency**	**Reference**
Prolonged labor	34.7%	2.00	Cases = 110 Controls = 219	329	366	Meshesha et al. [16]
Gestational age	22.7%	2.21	Cases = 97 Controls = 194	291	323	Tasew et al. [21]
Birth weight	9.3%	2.87	Cases = 94 Controls = 187	281	312	Mulugeta et al. [22]
Mode of delivery	7.8%	3.03	Cases = 96 Controls = 192	288	320	Meshesha et al. [16]
Parity	36.4%	3.103	Cases = 43 Controls = 86	129	143	Tasew et al. [21]

Cases were selected using a consecutive sampling technique until the required sample size was reached. Two controls per case were then selected through systematic random sampling. The sampling interval was calculated by dividing the average total number of nonasphyxiated newborns admitted over the previous 4 months by the required number of controls for the study.

### 2.4. Data Collection Procedure and Quality Control

Data was collected using a pretested, structured, interviewer‐administered questionnaire and a data abstraction checklist. Information on intrapartum and neonatal factors were gathered using the checklist, while maternal sociodemographic and antepartum characteristics were collected through the questionnaire. The data collection tools were adapted from relevant literature [4–6, 16, 17, 21–26] and modified to suit the study context.

The questionnaire was first prepared in English and then translated into the local languages, Amharic and Afan Oromo, with back‐translation to ensure accuracy. The English version of the checklist was used to collect data from medical records. Before the main data collection, a pretest was conducted on 5% of the sample to verify clarity and reliability.

Data collectors and supervisors received 2 days of training, covering the study′s purpose, participant approach, item‐by‐item clarification on the questionnaire, the data collection process, appropriate data source selection, and effective data and time management. Regular meetings and continuous supervision were implemented to maintain quality and consistency throughout the data collection process.

### 2.5. Data Processing and Statistical Analysis

Statistical Package for Social Sciences (SPSS) Version 27 was used to recode, clean, and analyze the data. Descriptive statistics summarized key characteristics of the study participants. A binary logistic regression model was applied to identify significant determinants of birth asphyxia. Variables with a *p* value below 0.25 in the bivariable analysis were selected for inclusion in the multivariable binary logistic regression analysis to adjust for potential confounders. The model was developed using a standard model‐building approach, and its fit was assessed using Hosmer and Lemeshow′s goodness‐of‐fit test; the result showed a good fit. Multicollinearity among the explanatory variables was checked using the variance inflation factor (VIF) and found to be within acceptable limits. In the final model, adjusted odds ratios (AORs) with 95% confidence intervals (CIs) were reported, and variables with a *p* value < 0.05 were considered statistically significant.

## 3. Result

### 3.1. Sociodemographic Characteristics

A total of 345 participants (115 cases and 230 controls) took part in the study, resulting in a response rate of 94.26%. The median age of mothers was 26 years (IQR: 23–30) for cases and 26 years (IQR: 23–29) for controls. Most participants were married, with 105 cases (91.3%) and 206 controls (89.6%) reporting being married. The majority of cases, 74 (64.3%), and nearly half of the controls, 99 (43.0%), were rural residents. Among the cases, 82 (71.3%) were housewives, compared to 124 (43.9%) among the controls (Table 2).

**Table 2:** Sociodemographic characteristics of the study participants.

**Variables**	**Cases (%)**	**Control (%)**
Age of the mother (in years)		
15–19	7 (6.1%)	3 (1.3%)
20–24	91 (79.1%)	209 (90.9%)
> 35	17 (14.8%)	18 (7.8%)
Marital status		
Unmarried	10 (8.7%)	24 (10.4%)
Married	105 (91.3%)	206 (89.6%)
Residence		
Rural	74 (64.3%)	99 (43.0%)
Urban	41 (35.7%)	131 (57.0%)
Ethnicity		
Oromo	92 (80.0%)	156 (67.8%)
Amhara	18 (15.7%)	60 (26.1%)
Others^a^	5 (4.3%)	14 (6.1%)
Educational status		
No formal education	20 (17.4%)	23 (12.9%)
Primary	17 (14.8%)	56 (31.5%)
Secondary	38 (33.0)	53 (29.8%)
College and above	40 (34.8%)	46 (25.8%)
Mother′s occupation		
Housewife	82 (71.3%)	124 (53.9%)
Government employee	18 (15.7%)	44 (19.1%)
Merchant	9 (7.8%)	43 (18.7%)
Others^b^	6 (5.2%)	19 (8.3%)
Monthly income (average in ETB)		
≤ 2000	76 (66.1%)	126 (54.8%)
2001–5000	29 (25.2%)	72 (31.3%)
˃ 5000	10 (8.7%)	32 (13.9%)

^a^Wolaita and Gurage.

^b^Daily labor and nongovernmental organization employee.

### 3.2. Obstetrics‐Related Characteristics

In this study, 97 cases (84.3%) and 211 controls (91.7%) had ANC follow‐up. Approximately half of the cases (50.4%) and 78.7% of the controls gave birth via spontaneous vaginal delivery. Among the cases, 96 (83.5%) experienced prolonged labor, compared to 15 (6.5%) of the controls. Premature rupture of membranes occurred in 22 cases (19.1%) and 21 controls (9.1%). Additionally, nine cases (7.8%) and 29 controls (12.6%) had anemia during pregnancy (Table 3).

**Table 3:** Obstetrics‐related characteristics of the study participants.

**Variables**	**Cases (%)**	**Control (%)**
ANC follow‐up		
Yes	97 (84.3%)	211 (91.7%)
No	18 (15.7%)	19 (8.3%)
Number of ANC visits		
1–3	80 (82.5%)	185 (87.7%)
≥ 4	17 (17.5%)	26 (11.3%)
Parity		
Primipara	58 (50.4%)	101 (43.9%)
Multipara	57 (49.6%)	129 (56.1%)
Birth space (for multipara)		
< 2 years	18 (31.6%)	41 (31.8%)
≥ 2 years	39 (68.4%)	88 (68.2%)
Mode of delivery		
Spontaneous vaginal delivery	58 (50.4%)	181 (78.7%)
Instrumental delivery	22 (19.1%)	19 (8.3%)
Cesarean section delivery	35 (30.4%)	30 (13.0%)
Type of anesthesia for cesarean section delivery		
Spinal anesthesia	33 (94.3%)	24 (80.0%)
General anesthesia	2 (5.7%)	6 (20.0%)
Birth attendant		
Midwife	46 (40.0%)	172 (74.8%)
Doctor	39 (33.9%)	32 (13.9%)
Integrated emergency surgical officer (IESO)	30 (26.1%)	26 (11.3%)
Amniotic fluid		
Clear	3 (2.6%)	192 (83.5%)
Meconium stained	112 (97.4%)	38 (16.5%)
Type of labor		
Spontaneous	97 (84.3%)	187 (81.3%)
Induced	18 (15.7%)	43 (18.7%)
Prolonged labor		
Yes	66 (57.4%)	25 (10.9%)
No	49 (42.6%)	205 (89.1%)
Premature rupture of membrane		
Yes	22 (19.1%)	21 (9.1%)
No	93 (80.9%)	209 (90.9%)
Cord prolapse		
Yes	7 (6.1%)	3 (1.3%)
No	108 (93.9%)	227 (98.7%)
Chorioamnionitis		
Yes	16 (13.9%)	13 (5.7%)
No	99 (86.1%)	217 (94.3%)
Preeclampsia/eclampsia		
Yes	22 (19.1%)	23 (10.0%)
No	93 (80.9%)	207 (90.0%)
Antepartum hemorrhage		
Yes	17 (14.8%)	13 (5.7%)
No	98 (85.2%)	217 (94.3%)
Anemia during pregnancy		
Yes	9 (7.8%)	29 (12.6%)
No	106 (92.2%)	201 (87.4%)

Abbreviation: ANC, antenatal care.

### 3.3. Newborn‐Related Characteristics

This study showed that 62 cases (53.9%) and 136 controls (59.1%) were male newborns. Ninety‐four cases (81.7%) and 221 controls (96.1%) were term newborns. The majority, 81 cases (70.4%) and 209 controls (90.9%), had a normal birth weight (Table 4).

**Table 4:** Newborn‐related characteristics of the study participants.

**Variables**	**Cases (%)**	**Control (%)**
Sex of the newborn		
Male	62 (53.9%)	136 (59.1%)
Female	53 (46.1%)	94 (40.9%)
Gestational age		
Preterm	16 (13.9%)	5 (2.2%)
Term	94 (81.7%)	221 (96.1%)
Postterm	5 (4.3%)	4 (1.7%)
Birth weight		
Low birth weight	30 (26.1%)	17 (7.4%)
Normal	81 (70.4%)	209 (90.9%)
Macrosomia	4 (3.5%)	4 (1.7%)
Type of birth		
Singleton	95 (82.6%)	216 (93.9%)
Twin	20 (17.4%)	14 (6.1%)
Fetal presentation		
Cephalic	97 (84.3%)	215 (93.5%)
Noncephalic	18 (15.7%)	15 (6.5%)
Able to cry at birth		
Yes	5 (4.3%)	207 (90.0%)
No	110 (95.7%)	23 (10.0%)

### 3.4. Determinants of Birth Asphyxia

In the bivariable binary logistic regression analysis, factors such as maternal age, residence, premature rupture of membrane, preeclampsia/eclampsia, prolonged labor, mode of delivery, fetal presentation, gestational age, and birth weight showed statistically significant associations with birth asphyxia at a *p* value of < 0.25. After adjusting for potential confounders in a multivariable logistic regression analysis, residence, premature rupture of membrane, prolonged labor, mode of delivery, and fetal presentation remained independent determinants of birth asphyxia at a *p* value of < 0.05.

Thus, the odds of birth asphyxia were 2.3 times higher among newborns born to mothers residing in rural areas compared to those in urban areas (AOR = 2.34; 95% CI: 1.29–4.26). Newborns whose mothers experienced premature rupture of membranes had 3.5 times higher odds of developing birth asphyxia compared to their counterparts (AOR = 3.47; 95% CI: 1.52–7.92). Newborns whose mothers underwent prolonged labor had tenfold greater odds of experiencing birth asphyxia compared to those whose mothers did not have prolonged labor (AOR = 10.12; 95% CI: 5.36–19.11). Compared to newborns born with cephalic presentation, those born with noncephalic presentation had twice the odds of facing birth asphyxia (AOR = 2.40; 95% CI: 1.01–5.74). Moreover, the odds of birth asphyxia were 2.7 times higher (AOR = 2.67; 95% CI: 1.15–6.16) and four times higher (AOR = 3.99; 95% CI: 1.84–8.63) among newborns born via instrumental delivery and cesarean section, respectively, compared to those born via spontaneous vaginal delivery (Table 5).

**Table 5:** Determinants of birth asphyxia among newborns at a tertiary care hospital, Central Ethiopia.

**Variables**	**Birth asphyxia status**		**COR (95% CI)**	**AOR (95% CI)**	**p** **value**
	**Cases (%)**	**Controls (%)**			
Age of the mother (in years)					
20–24	91 (79.1)	209 (90.9)	1	1	
15–19	7 (6.1)	3 (1.3)	5.36 (1.36, 21.19) ^∗^	3.63 (0.85, 15.41)	0.081
> 35	17 (14.8)	18 (7.8)	2.17 (1.07, 4.40)	2.14 (0.92, 4.99)	0.078
Residence					
Rural	74 (64.3)	99 (43.0)	2.39 (1.49–3.75) ^∗^	2.34 (1.29–4.26) ^∗∗^	0.005
Urban	41 (35.7)	131 (57.0)	1	1	
Premature rupture of membrane					
Yes	22 (19.1)	21 (9.1)	2.35 (1.23–4.49) ^∗^	3.47 (1.52–7.92) ^∗∗^	0.003
No	93 (80.9)	209 (90.9)	1	1	
Preeclampsia/eclampsia					
Yes	22 (19.1)	23 (10.0)	2.13 (1.15–36.16) ^∗^	5.64 (0.81–39.21)	0.081
No	93 (80.9)	207 (90.0)	1	1	
Prolonged labor					
Yes	66 (57.4)	25 (10.9)	11.05 (4.52–12.29) ^∗^	10.12 (5.36–19.11) ^∗∗^	< 0.001
No	49 (42.6)	205 (89.1)	1	1	
Fetal presentation					
Cephalic	97 (84.3)	215 (93.5)	1	1	
Noncephalic	18 (15.7)	15 (6.5)	2.66 (1.29–5.50) ^∗^	2.40 (1.01–5.74) ^∗∗^	0.048
Mode of delivery					
Spontaneous vaginal delivery	58 (50.4)	181 (78.7)	1	1	
Instrumental delivery	22 (19.1)	19 (8.3)	3.63 (1.84–7.18) ^∗^	2.67 (1.15–6.16) ^∗∗^	0.022
Cesarean section delivery	35 (30.4)	30 (13.0)	3.66 (2.07–6.48) ^∗^	3.99 (1.84–8.63) ^∗∗^	< 0.001
Gestational age					
Term	94 (81.7)	221 (96.1)	1	1	
Preterm	16 (13.9)	5 (2.2)	7.56 (2.69–21.23) ^∗^	5.34 (0.98–19.70)	0.209
Postterm	5 (4.3)	4 (1.7)	2.95 (0.78–11.24)	2.87 (0.54–15.25)	0.216
Birth weight					
Low birth weight	30 (26.1)	17 (7.4)	1.76 (1.39–4.74) ^∗^	1.69 (0.89–6.85)	0.314
Normal birth weight	81 (74.3)	209 (90.9)	0.39 (0.63–10.61)	4.37 (0.72–26.53)	0.109
Macrosomia	4 (3.5)	4 (1.7)	1	1	

*Note:* 1 = reference.

Abbreviations: AOR, adjusted odds ratio; CI, confidence interval; COR, crude odds ratio.

^∗^Significant at *p* value < 0.25 in unadjusted logistic regression analysis.  ^∗∗^Significant at *p* < 0.05 in adjusted logistic regression analysis.

## 4. Discussion

The current study was aimed at identifying the determinants of birth asphyxia among newborns at a tertiary care hospital in Central Ethiopia. In this study, we identified residence, premature rupture of membranes, prolonged labor, mode of delivery, and fetal presentation as the independent determinants of birth asphyxia.

This study revealed that newborns born to mothers residing in rural areas had higher odds of experiencing birth asphyxia compared to those in urban areas. This finding is concurrent with those of studies conducted in Nepal [19], Tanzania [6], and Ethiopia [5, 27]. A possible explanation could be the limited access to quality antenatal and prenatal care in rural areas, compounded by challenges such as the unavailability and distance of healthcare facilities, inadequate awareness about the importance of ANC, transportation difficulties, and the heavy burden of household responsibilities on rural women.

Newborns whose mothers experienced premature rupture of membranes had increased odds of developing birth asphyxia compared to their counterparts. This finding is in line with previous studies conducted in Nepal [19], Saudi Arabia [28], Cameroon [29], and Ethiopia [5, 15, 24, 27, 30]. After premature rupture of membranes, the amniotic fluid no longer cushions the umbilical cord, increasing the risk of cord compression. Such compression can interrupt the supply of oxygen‐rich blood to the baby, potentially causing birth asphyxia. Additionally, the loss of the amniotic sac′s protective barrier may allow maternal infections to reach the baby more easily, potentially leading to sepsis and brain damage, both of which can contribute to birth asphyxia [2, 31, 32].

Consistent with previous studies done in Sweden [33], Pakistan [34], Rwanda [25], Cameroon [29], Tanzania [6], and Ethiopia [5, 16–18, 26, 35–37], this study showed that newborns whose mothers underwent prolonged labor had tenfold greater odds of experiencing birth asphyxia compared to those whose mothers did not have prolonged labor. This can be justified by the fact that prolonged labor increases the risk of birth asphyxia due to potential complications such as uterine exhaustion, reduced placental blood flow, and fetal distress. These conditions can lead to insufficient oxygen supply to the fetus during labor, contributing to birth asphyxia. Additionally, prolonged labor may increase the likelihood of medical interventions or infections, further exacerbating the risk [38, 39].

The current study also revealed that newborns with noncephalic presentations had twice the odds of experiencing birth asphyxia compared to those with cephalic presentations. The finding is in line with previous studies conducted in Pakistan [20], Cameroon [29], and Ethiopia [4, 17, 18, 30, 35, 37, 40]. A possible explanation is that noncephalic presentations can lead to complications such as umbilical cord prolapse or compression, which can significantly impair oxygen supply to the fetus, causing hypoxia. Additionally, noncephalic presentations may be associated with fetal distress, as evidenced by altered fetal heart rates, decreased fetal movements, and the presence of meconium‐stained amniotic fluid, all of which increase the likelihood of birth asphyxia [41, 42].

The mode of delivery was another significant determinant of birth asphyxia. The odds of birth asphyxia were higher among newborns delivered via instrumental delivery and cesarean section compared to those delivered through spontaneous vaginal delivery. This finding is supported by previous studies conducted in Nepal [43], Indonesia [44], Tanzania [6], and Ethiopia [4, 5, 16, 22, 23, 26, 27, 35, 37, 45, 46]. This may be because these delivery methods are often employed in high‐risk situations, such as fetal distress or prolonged labor, where oxygen deprivation may already be present. Furthermore, the mechanical interventions during instrumental deliveries and the delays or complications associated with emergency cesarean sections can exacerbate fetal oxygen deprivation, thereby increasing the risk of birth asphyxia.

When interpreting the study′s findings, it is important to acknowledge certain limitations. The study was an institution‐based study where most births were attended by qualified health professionals, which may not accurately represent risk factors at the community level. Additionally, the inherent constraints of the case‐control design limit the ability to establish causal relationships.

## 5. Conclusion

In conclusion, we identified rural residence, premature rupture of membranes, prolonged labor, mode of delivery (including instrumental delivery and cesarean section), and noncephalic fetal presentation as independent determinants of birth asphyxia. Based on these findings, we recommend improving access to quality obstetric care in rural areas, strengthening early detection and management of prolonged labor and premature rupture of membranes, and ensuring skilled decision‐making during delivery, particularly for noncephalic presentations and instrumental or cesarean deliveries.

**Nomenclature**
AOR: adjusted odds ratio
ANC: antenatal care
CIs: confidence intervals
COR: crude odds ratio
OR: odds ratio
SPSS: Statistical Package for Social Sciences

## Ethics Statement

Ethical clearance was secured from the Institutional Review Board of Adama Hospital Medical College. A permission letter was submitted to the hospital administration, which subsequently granted approval to access medical records and conduct the study. Mothers were thoroughly informed about the study′s potential benefits and risks, and their voluntary participation or refusal was documented through written consent. Anonymity and privacy were safeguarded throughout the study to protect participants′ rights and maintain confidentiality. All procedures adhered to the principles outlined in the Helsinki Declaration.

## Consent

The authors have nothing to report.

## Disclosure

All authors read and approved the final manuscript. This study was posted as a preprint. This study has also been posted as a preprint [47].

## Conflicts of Interest

The authors declare no conflicts of interest.

## Author Contributions

T.A. contributed to the design, data curation, and supervision. N.M.F. and Y.M.N. worked on the formal analysis and methodology and drafted the manuscript. Y.M.N. critically reviewed the draft manuscript and wrote the final version. A.M.A., A.D., A.T.C., and E.B.W. advised the study.

## Funding

No funding was received for this manuscript.

## Data Availability

All data and materials are available from the corresponding author without undue reservation.

## References

[1] Golubnitschaja O. , Yeghiazaryan K. , Cebioglu M. , Morelli M. , and Herrera-Marschitz M. , Birth Asphyxia as the Major Complication in Newborns: Moving Towards Improved Individual Outcomes by Prediction, Targeted Prevention and Tailored Medical Care, EPMA Journal. (2011) 2, no. 2, 197–210, 10.1007/s13167-011-0087-9, 2-s2.0-79961043439, 23199149. 23199149 PMC3405378

[2] Gillam-Krakauer M. , Shah M. , and Gowen C. W.Jr., Birth Asphyxia, StatPearls, 2024, StatPearls Publishing. 28613533

[3] Perinatal Asphyxia, accessed November 19, 2024, https://www.who.int/teams/maternal-newborn-child-adolescent-health-and-ageing/newborn-health/perinatal-asphyxia.

[4] Lemma K. , Misker D. , Kassa M. , Abdulkadir H. , and Otayto K. , Determinants of Birth Asphyxia Among Newborn Live Births in Public Hospitals of Gamo and Gofa Zones, Southern Ethiopia, BMC Pediatrics. (2022) 22, no. 1, 10.1186/s12887-022-03342-x, 35562670. PMC9099035 35562670

[5] Ahmed R. , Mosa H. , Sultan M. , Helill S. E. , Assefa B. , Abdu M. , Ahmed U. , Abose S. , Nuramo A. , Alemu A. , Demelash M. , and Delil R. , Prevalence and Risk Factors Associated With Birth Asphyxia Among Neonates Delivered in Ethiopia: A Systematic Review and Meta-Analysis, PLoS One. (2021) 16, no. 8, 10.1371/journal.pone.0255488. PMC8341515 34351953

[6] Msisiri L. S. , Kibusi S. M. , and Kimaro F. D. , Risk Factors for Birth Asphyxia in Hospital-Delivered Newborns in Dodoma, Tanzania: A Case-Control Study, SAGE Open Nursing. (2024) 10, 10.1177/23779608241246874, 38665876. PMC11044786 38665876

[7] Wallander J. L. , Bann C. , Chomba E. , Goudar S. S. , Pasha O. , Biasini F. J. , McClure E. M. , Thorsten V. , Wallace D. , and Carlo W. A. , Developmental Trajectories of Children With Birth Asphyxia Through 36 Months of Age in Low/Low–Middle Income Countries, Early Human Development. (2014) 90, no. 7, 343–348, 10.1016/j.earlhumdev.2014.04.013, 2-s2.0-84925225924, 24815056. 24815056 PMC4097313

[8] Van Bel F. and Groenendaal F. , Birth Asphyxia-Induced Brain Damage: The Long Road to Optimal Reduction and Prevention!, Pediatric Medicine. (2020) 3, 10.21037/pm.2019.11.02.

[9] Workineh Y. , Semachew A. , Ayalew E. , Animaw W. , Tirfie M. , and Birhanu M. , Prevalence of Perinatal Asphyxia in East and Central Africa: Systematic Review and Meta-Analysis, Heliyon. (2020) 6, no. 4, e03793, 10.1016/j.heliyon.2020.e03793, 32368646. 32368646 PMC7184262

[10] Grady S. C. , Frake A. N. , Zhang Q. , Bene M. , Jordan D. R. , Vertalka J. , Dossantos T. C. , Kadhim A. , Namanya J. , Pierre L.-M. , Fan Y. , Zhou P. , Barry F. B. , and Kutch L. , Neonatal Mortality in East Africa and West Africa: A Geographic Analysis of District-Level Demographic and Health Survey Data, Geospatial Health. (2017) 12, no. 1, 10.4081/gh.2017.501, 2-s2.0-85021074429, 28555482. 28555482

[11] Mekonnen W. , Assefa N. , Asnake W. , Sahile Z. , and Hailemariam D. , Under Five Causes of Death in Ethiopia Between 1990 and 2016: Systematic Review With Meta-Analysis, Ethiopian Journal of Health Development. (2020) 34, no. 2.

[12] Manandhar M. , Hawkes S. , Buse K. , Nosrati E. , and Magar V. , Gender, Health and the 2030 Agenda for Sustainable Development, Bulletin of the World Health Organization. (2018) 96, no. 9, 644–653, 10.2471/BLT.18.211607, 2-s2.0-85054051950, 30262946. 30262946 PMC6154065

[13] Health Sector Transformation Plan II (HSTP II) 2020/21-2024/25 | Country Planning Cycle Database, accessed November 21, 2024, https://extranet.who.int/countryplanningcycles/planning-cycle-files/health-sector-transformation-plan-ii-hstp-ii-202021-202425.

[14] Antonucci R. , Porcella A. , and Pilloni M. D. , Perinatal Asphyxia in the Term Newborn, Journal of Pediatric and Neonatal Individualized Medicine. (2014) 3, no. 2, e030269.

[15] Woday A. , Muluneh A. , and St Denis C. , Birth Asphyxia and Its Associated Factors Among Newborns in Public Hospital, Northeast Amhara, Ethiopia, PLoS One. (2019) 14, no. 12, e0226891, 10.1371/journal.pone.0226891, 31860643. 31860643 PMC6924666

[16] Meshesha A. D. , Azage M. , Worku E. , and Bogale G. G. , Determinants of Birth Asphyxia Among Newborns in Referral Hospitals of Amhara National Regional State, Ethiopia, Pediatric Health, Medicine and Therapeutics. (2020) 11, 1–12, 10.2147/PHMT.S229227, 32021550. 32021550 PMC6954855

[17] Tegegnework S. S. , Gebre Y. T. , Ahmed S. M. , and Tewachew A. S. , Determinants of Birth Asphyxia Among Newborns in Debre Berhan Referral Hospital, Debre Berhan, Ethiopia: A Case-Control Study, BMC Pediatrics. (2022) 22, no. 1, 10.1186/s12887-022-03223-3, 35354399. PMC8966276 35354399

[18] Fekede T. and Fufa A. , Determinants of Birth Asphyxia at Public Hospitals in Ilu Aba Bor Zone Southwest, Ethiopia: A Case Control Study, Scientific Reports. (2022) 12, no. 1, 10705, 10.1038/s41598-022-15006-y, 35739178. 35739178 PMC9226011

[19] Rijal M. , Chaudhary T. K. , and Saha A. , Determinants of Birth Asphyxia Among Newborn in a Zonal Hospital, Janaki Medical College Journal of Medical Science. (2023) 11, no. 2, 28–39, 10.3126/jmcjms.v11i2.58026.

[20] Aslam H. M. , Saleem S. , Afzal R. , Iqbal U. , Saleem S. M. , Shaikh M. W. A. , and Shahid N. , Risk Factors of Birth Asphyxia, Italian Journal of Pediatrics. (2014) 40, no. 1, 10.1186/s13052-014-0094-2, 2-s2.0-84965084751. PMC4300075 25526846

[21] Tasew H. , Zemicheal M. , Teklay G. , Mariye T. , and Ayele E. , Risk Factors of Birth Asphyxia Among Newborns in Public Hospitals of Central Zone, Tigray, Ethiopia 2018, BMC Research Notes. (2018) 11, no. 1, 10.1186/s13104-018-3611-3, 2-s2.0-85050387284, 30029614. PMC6053756 30029614

[22] Mulugeta T. , Sebsibe G. , Fenta F. A. , and Sibhat M. , Risk Factors of Perinatal Asphyxia Among Newborns Delivered at Public Hospitals in Addis Ababa, Ethiopia: Case–Control Study, Pediatric Health, Medicine and Therapeutics. (2020) 11, 297–306, 10.2147/PHMT.S260788, 32922119. 32922119 PMC7457880

[23] Lake E. S. , Abita Z. , and Erega B. B. , Determinants of Birth Asphyxia Among Newborns in South Gondar Zone Public Hospitals, North West Ethiopia, 2021: A Case Control Study, Heliyon. (2024) 10, no. 9, e30093, 10.1016/j.heliyon.2024.e30093, 38707282. 38707282 PMC11068594

[24] Tunta T. , Dana T. , Wolie A. , and Lera T. , Determinants of Birth Asphyxia Among Neonates Admitted to Neonatal Intensive Care Units in Hospitals of the Wolaita Zone, Southern Ethiopia: A Case-Control Study, Heliyon. (2024) 10, no. 1, e23856, 10.1016/j.heliyon.2023.e23856, 38192802. 38192802 PMC10772715

[25] Theoneste N. and Angelique M. , Factors Contributing to Birth Asphyxia as the Major Complication Among Newborns Delivered at Gitwe District Hospital, Southern Province, Rwanda, International Journal of Public Health and Clinical Sciences. (2017) 4, no. 3, 142–151.

[26] Wosenu L. , Worku A. G. , Teshome D. F. , and Gelagay A. A. , Determinants of Birth Asphyxia Among Live Birth Newborns in University of Gondar Referral Hospital, Northwest Ethiopia: A Case-Control Study, PLoS One. (2018) 13, no. 9, e0203763, 10.1371/journal.pone.0203763, 2-s2.0-85053111958, 30192884. 30192884 PMC6128623

[27] Admasu F. T. , Melese B. D. , Amare T. J. , Zewude E. A. , Denku C. Y. , and Dejenie T. A. , The Magnitude of Neonatal Asphyxia and Its Associated Factors Among Newborns in Public Hospitals of North Gondar Zone, Northwest Ethiopia: A Cross-Sectional Study, PLoS One. (2022) 17, no. 3, e0264816, 10.1371/journal.pone.0264816, 35245309. 35245309 PMC8896710

[28] Sahib H. S. , Risk Factors of Perinatal Asphyxia: A Study at Al-Diwaniya Maternity and Children Teaching Hospital, Muthanna Medical Journal. (2015) 2, no. 2, 50–57.

[29] Chiabi A. , Nguefack S. , Mah E. , Nodem S. , Mbuagbaw L. , Mbonda E. , Tchokoteu P.-F. , and Doh Frcog A. , Risk Factors for Birth Asphyxia in an Urban Health Facility in Cameroon, Iranian Journal of Child Neurology. (2013) 7, no. 3, 46–54, 24665306. 24665306 PMC3943072

[30] Bayih W. A. , Yitbarek G. Y. , Aynalem Y. A. , Abate B. B. , Tesfaw A. , Ayalew M. Y. , Belay D. M. , Hailemeskel H. S. , and Alemu A. Y. , Prevalence and Associated Factors of Birth Asphyxia Among Live Births at Debre Tabor General Hospital, North Central Ethiopia, BMC Pregnancy and Childbirth. (2020) 20, no. 1, 10.1186/s12884-020-03348-2, 33115413. PMC7594464 33115413

[31] Dayal S. , Jenkins S. M. , and Hong P. L. , Preterm and Term Prelabor Rupture of Membranes (PPROM and PROM), StatPearls, 2024, StatPearls Publishing. 30422483

[32] Boushra M. , Stone A. , and Rathbun K. M. , Umbilical Cord Prolapse, StatPearls, 2024, StatPearls Publishing. 31194398

[33] Sandström A. , Altman M. , Cnattingius S. , Johansson S. , Ahlberg M. , and Stephansson O. , Durations of Second Stage of Labor and Pushing, and Adverse Neonatal Outcomes: A Population-Based Cohort Study, Journal of Perinatology. (2017) 37, no. 3, 236–242, 10.1038/jp.2016.214, 2-s2.0-85001832923, 27929527. 27929527 PMC5339416

[34] Nadeem G. , Rehman A. , and Bashir H. , Risk Factors Associated With Birth Asphyxia in Term Newborns at a Tertiary Care Hospital of Multan, Pakistan, Cureus. (2021) 13, no. 10, e18759, 10.7759/cureus.18759, 34796056. 34796056 PMC8590025

[35] Wayessa Z. , Belachew T. , and Joseph J. , Birth Asphyxia and Associated Factors Among Newborns Delivered in Jimma Zone Public Hospitals, Southwest Ethiopia: A Cross-Sectional Study, Journal of Midwifery & Reproductive Health. (2018) 6, no. 2.

[36] Abdo R. A. , Halil H. M. , Kebede B. A. , Anshebo A. A. , and Gejo N. G. , Prevalence and Contributing Factors of Birth Asphyxia Among the Neonates Delivered at Nigist Eleni Mohammed Memorial Teaching Hospital, Southern Ethiopia: A Cross-Sectional Study, BMC Pregnancy and Childbirth. (2019) 19, no. 1, 10.1186/s12884-019-2696-6, 31888542. PMC6937931 31888542

[37] Alamneh Y. M. , Negesse A. , Aynalem Y. A. , Shiferaw W. S. , Gedefew M. , Tilahun M. , Hune Y. , Abebaw A. , Biazin Y. , and Akalu T. Y. , Risk Factors of Birth Asphyxia Among Newborns at Debre Markos Comprehensive Specialized Referral Hospital, Northwest Ethiopia: Unmatched Case-Control Study, Ethiopian Journal of Health Sciences. (2022) 32, no. 3, 513–522, 10.4314/ejhs.v32i3.6, 35813672. 35813672 PMC9214735

[38] Mota-Rojas D. , Villanueva-García D. , Solimano A. , Muns R. , Ibarra-Ríos D. , and Mota-Reyes A. , Pathophysiology of Perinatal Asphyxia in Humans and Animal Models, Biomedicine. (2022) 10, no. 2, 10.3390/biomedicines10020347. PMC8961792 35203556

[39] Lawn J. E. , Blencowe H. , Oza S. , You D. , Lee A. C. , Waiswa P. , Lalli M. , Bhutta Z. , Barros A. J. , Christian P. , Mathers C. , Cousens S. N. , and Lancet Every Newborn Study Group , Every Newborn: Progress, Priorities, and Potential Beyond Survival, Lancet. (2014) 384, no. 9938, 189–205, 10.1016/S0140-6736(14)60496-7, 2-s2.0-84904185064, 24853593. 24853593

[40] Berhe Y. Z. , Kebedom A. G. , Gebregziabher L. , Assefa N. E. , Berhe L. Z. , Mohammednur S. A. , Wellay T. , Berihu G. , Welearegay A. T. , Mitiku M. , and Teka H. G. , Risk Factors of Birth Asphyxia Among Neonates Born in Public Hospitals of Tigray, Northern Ethiopia, Pediatric Health, Medicine and Therapeutics. (2020) 11, 13–20, 10.2147/PHMT.S231290, 32021551. 32021551 PMC6955612

[41] Nicholson J. M. , Non-Cephalic Presentation in Late Pregnancy, BMJ. (2006) 333, no. 7568, 562–563, 10.1136/bmj.38971.476863.AB, 2-s2.0-33748928225, 16973991. 16973991 PMC1569998

[42] Martel-Santiago C. R. , Arencibia-Díaz R. D. , Romero-Requejo A. , Valle-Morales L. , Figueras-Falcón T. , García-Hernández J. Á. , and Martin-Martinez A. , Delivery in Breech Presentation: Perinatal Outcome and Neurodevelopmental Evaluation at 18 Months of Life, European Journal of Obstetrics & Gynecology and Reproductive Biology. (2020) 255, 147–153, 10.1016/j.ejogrb.2020.10.020, 33130377. 33130377

[43] Sunny A. K. , Paudel P. , Tiwari J. , Bagale B. B. , Kukka A. , Hong Z. , Ewald U. , Berkelhamer S. , and Kc A. , A Multicenter Study of Incidence, Risk Factors and Outcomes of Babies With Birth Asphyxia in Nepal, BMC Pediatrics. (2021) 21, no. 1, 10.1186/s12887-021-02858-y, 34507527. PMC8431921 34507527

[44] Kardana I. M. , Risk Factors of Perinatal Asphyxia in the Term Newborn at Sanglah General Hospital, Bali-Indonesia, Bali Medical Journal. (2016) 5, no. 1, 10.15562/bmj.v5i1.312.

[45] Amare Wudu M. and Birehanu T. A. , Predictors of Birth Asphyxia Among Newborns in Public Hospitals of Eastern Amhara Region, Northeastern Ethiopia, 2022, Clinical Medicine Insights: Pediatrics. (2023) 17, 10.1177/11795565231196764, 37719038. PMC10504851 37719038

[46] Bayih W. A. , Birhane B. M. , Belay D. M. , Ayalew M. Y. , Yitbarek G. Y. , Workie H. M. , Abie Tassew D. M. , Kebede S. D. , Alemu A. Y. , Gedefaw G. , Demis A. , and Chanie E. S. , The State of Birth Asphyxia in Ethiopia: An Umbrella Review of Systematic Review and Meta-Analysis Reports, 2020, Heliyon. (2021) 7, no. 10, e08128, 10.1016/j.heliyon.2021.e08128, 34746456. 34746456 PMC8551510

[47] Alemayehu T. , Fati N. M. , Megerso A. , and Deksisa A. , Determinants of Birth Asphyxia Among Newborns in Adama Hospital Medical College, Adama Town, Oromia Region, Ethiopia: A Case Control Study, 2023, https://www.researchsquare.com/article/rs-3523310/v1.

